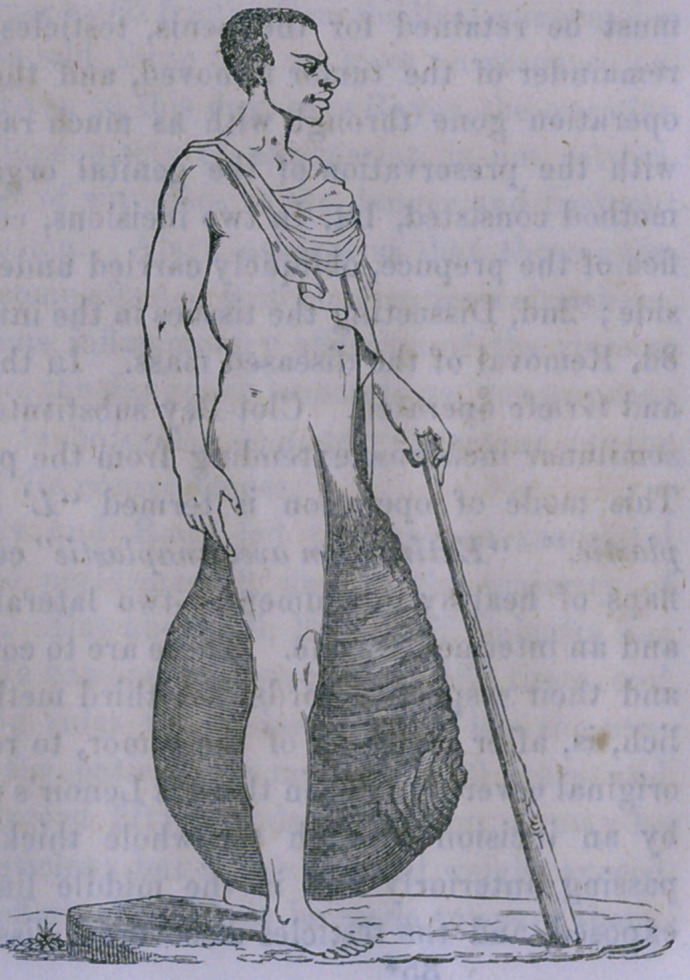# Book and Pamphlet Notices

**Published:** 1859-09

**Authors:** 


### BOOK AND PAMPHLET NOTICES.

Rapport a la Societe de Chirurgie sur L' Elephantiasis du Scrotum. Par M. Hte. Bon. Larrey, Chirurgien ordinaire de l' Empereur, Medecin en chef, etc., etc.

Report on Elephantiasis of the Scrotum, made to the Society of Surgery. By M. Bon. Hte. Larrey, Surgeon in Ordinary to his Majesty the Emperor. Paris. 1856.

This we have no hesitation in pronouncing the best monograph on this disease ever issued from the press.

A memoir, presented to the Societe de Chirurgie, Paris, by the celebrated Clot-Bey, of Egypt, was referred to a commission, of which M. Larrey was the reporter. For the purpose of studying the subject fully, he was induced to enter upon inquiries that have amply furnished him with materials, of which this work is a digest.

He commences with some reflections on the nature of elephantiasis generally, and shows how it is to be distinguished from elephantiasis of the Greeks, or tubercular leprosy—an altogether distinct disease, and confounded with that under consideration, owing to the employment of a vicious nomenclature. He then views elephantiasis as it affects the scrotum; considers its causes, symptoms, complication, diagnosis, prognosis, treatment, terminations and morbid anatomy.

The writer has availed himself of every interesting case on record; and as many of these are scattered through journals under various names, we can well appreciate the trouble experienced and the perseverance required in first discovering and then divesting them of much that was calculated to obscure and confuse.

We find Dionis, Cheselden, Morgagni, and even the celebrated Larrey, surgeon-in-chief of the French army, employing, in reference to this disease, the term "sarcocele,"—a word without precise signification, and therefore leading to indistinct conceptions. Then we have had "*oscheochalasie*," proposed by Alibert, who afterwards, by way of improvement, substituted the not less objectionable word, oscheo-terastie-sarcomateux. Richerand, in his effort to improve on this, calls it "lymphatic tumor of the scrotum." Now this designation involves a theory; and in the present state of medical knowledge, nothing is more likely to lead to errors of practice than the adoption of a nomenclature resting for support on a questionable doctrine, or some fancied accordance with a speculation in science.

The writer affirms that no name hitherto applied succeeds in conveying correct notions either of the essential nature or anatomical seat of the disease. He thinks the disease, so frequently circumscribed and located in the scrotum, seems to find, in the mere texture of the parts, conditions of existence different from those that could belong exclusively to the lymphatic vessels. This he thinks countenanced by the fact that in elephantiasis confined to the scrotum (and without complication, we presume), inguinal adenitis almost never exists. He appeals to Cazenave, who, he says, "saw with Biett, even an example of elephantiasis of *a limb*, in which there was no sign of angio-leucitis;" and "Rayer," he adds, "had occasion still better to prove the absence of every trace of angio-leucitis." He denies the correctness of the opinion of Alard, who explains the share the lymphatic system has in its production, by supposing there is a rupture of the lymphatic vessels in the acute form of elephantiasis, and a feebleness of the absorbent in the chronic. He assures us this rupture has never been satisfactorily demonstrated; and besides, he cannot comprehend how infiltration of lymph could produce effusions so various,—serous fluid, gelatinous matter and deposits of fibrine, together with fatty matter, accompanied with transformations of cellular tissue and prodigious thickening of the cutis.

He equally rejects the opinion of the disease being caused by a morbid state of the venous system. The nature of elephantiasis scroti he thinks still undetermined; but if he were required to define it, he would say it was "a tumefaction of the scrotum, characterized by hypertrophy and induration of the skin, and subcutaneous cellular tissue, with alteration of the other coats of the testicles; these organs, for the most part, remaining healthy in the midst of the disease."

The endemic nature of elephantiasis next comes under review. The observations of Larrey (sen.) and Titley would lead us to believe that it is a malady of warm climates rather than of cold countries. It has been particularly noticed in Barbadoes, and extends along the whole eastern coast of South America. In the other islands of the West Indies it is not as frequently seen as in Barbadoes. In Brazil it is common. The medical officers in British India testify as to its frequency in Asia. Bengal, the banks of the Ganges, the Malabar coast, Madras, the island of Ceylon, Surinam, etc., are the localities where endemic influence, in its production, is best shown; and some even say that in Japan, one of every ten persons is thus afflicted. He oppugns the doctrine held by Wise and others, that a humid climate exerts a morbid influence in its development, by shewing the Mauritius, though possessing a remarkably dry atmosphere, to contain a great number of such cases.

In some parts of Africa, elephantiasis would seem to be scarcely known, whilst in others it prevails exceedingly. Passing over Algeria, where it is common along the sea coast, we recognize it more abundantly still in Egypt. There was gleaned the experience respecting it by M. Clot-Bey, the writer of the memoir, that led to this valuable report.

In Europe it is infinitely more rare than in the other great divisions of the globe. It is seen, though exceptionally, in some towns of Spain and Italy, near the coast; but such cases as are seen in Germany, Prussia, Sweden, etc., are probably imported. Titley thinks the disease endemic in some parts of Ireland; but we are disposed to think it as exceptional there as in the sister kingdom. In Rousillon in France, however, Delpech thought the malady by no means rare.

As the etiology of elephantiasis scroti is allowed by the writer to be "still obscure and very vague," we will not dwell on the several conjectures advanced as presumable causes, but proceed to its symptoms and complications.

We learn that its invasion is either acute or chronic, with, we suppose, as in other complaints, many intermediate degrees that obtain, as the symptoms are characterized by more or less acuteness. "Hendy, in the description of the disease, which he has studied more in a general than in a local point of view, assigns to its invasion well marked febrile precursive symptoms, with enlargement of the glands." "The swelling of the parts augments, according to him, with or without new paroxysms of fever." "Sometimes," says Delpech, "it is by the prepuce the disease begins, and it may remain there stationary for a longer or shorter time without making marked progress;" "or, rather, the entire of the skin of the penis becomes hypertrophied, and assumes a coriaceous indurated condition before reaching the scrotum." This case is not frequent. Larrey observes: "Sometimes from the lower part of the pubic region, corresponding to the mons veneris in the female, the swelling begins to extend over all the external genital organs, or perhaps in some one point of the scrotum a nucleus of induration forming, elephantiasis declares itself." This he thinks the most usual form. Again, it may be observed invading at once the whole of the scrotum. Though the writer says the disease may begin without appreciable symptoms, and this opinion is in accordance with the observations of Delpech and Hendy, still, "in most cases, the commencement of the attack is marked by a swelling of the scrotum, with fever and very intense inflammatory reaction." "The reaction ceases, but the swelling persists, and becomes more and more decided as induration makes its appearance. This state may remain stationary without sensible increase of volume, but it almost always makes continual progress,—sometimes slow, at other times rapid. The tumor distends, becoming thicker, rugous and brown; the testicles are forced back, so as no longer to be seen or felt, still they undergo no morbid change necessarily arising from elephantiasis. When the disease is of long standing, yellow and scaly crusts appear, which, being detached, leave ulcerated surfaces behind, discharging ichor."

As the disease advances, and the penis is removed from view, a provision is made for the passing of urine by the formation of a supplemental canal, as it were, formed at the expense of the skin originally covering the body of the penis. This is drawn down by the weight of the tumor, and thus forms a prolongation or gutter extending from the urethra, often for six, eight, or even ten inches, to the middle or inferior part of the swelling, where it ends in a sort of umbilicus or narrow vulva. In such cases, as "the urine cannot be projected from the surface, but flows *en nappe*," we should expect the usual amount of irritation to arise from its contact with the skin; yet experience does not establish the supposition. There would seem to be, to some extent, a capability of resistance acquired by the hypertrophied skin. "The color of the part is not always brown; its surface is frequently blue or livid, owing to a varicose state of the veins, the dilatation of which is excessive." In one case, we are told the veins were so large that one of them equaled the vena cava in diameter.

This condition of the veins so often met with induced many to consider a morbid state of the venous system as the efficient cause of elephantiasis.

"The tumor is very soft at first, yet unlike œdema, in not leaving after pressure the impression of the finger. It gradually acquires the hardness of leather." "The tumor is sometimes hard in certain points and soft in others, giving, as if, a false sense of fluctuation. Though frequently at the commencement there may have been augmented sensibility, yet in the advanced stages there is scarcely any in the tumor, but rather a sense of inconvenience from its weight and volume." Now this is important with a view to operation, and as regards the propriety of dispensing with the aid of chloroform, the depressing influence of which might be contra-indicated when profuse hemorrhage, amounting sometimes to several pounds of blood, constitutes the chief danger of extirpation. An interesting feature of the disease, noticed by Hendy and others, is the intermittent character of the fever, and the well-marked connection between its paroxysms and the progressive increase of the local affection.

Some false notions have been entertained respecting the condition of the testicles, and their capability of preserving their function unimpaired in elephantiasis scroti. We have in the report a case in point, and we give as much of it as bears on the question.

"An Arab of the valley of Chelif, 40 years of age, married, in 1830, a widow affected with syphilis, and shortly afterwards he had a running from the urethra and ulcers on the penis. These symptoms disappeared in 1830 without having received any treatment. A pustular eruption was the consequence. The patient, as is the custom of the Arabs, made ablutions of cold water to the genital organs, and from that time he saw the scrotum swell in proportion as paroxysms of intermittent fever seemed to augment the swelling.

"Ten years afterwards, he entered the hospital of Orleansville, with a syphilitic papular eruption, and a well-marked elephantiasis scroti. The tumor measured thirty-eight centimetres in height and thirty-six in breadth."

The morbid mass was removed by M. Mestre with the most favorable result, and the patient, we are told, resumed an intercourse with his wife, whom he had repudiated fourteen years previously, and with whom, during that time, there had been a suspension of conjugal relations. There can be no doubt, however, that inaction of organs for several years, together with the compression to which at the same time they are subjected, must, in many instances, lead to their atrophy.

M. Larrey devotes much space to the consideration of the complications met with in this disease. Without following him in all the details, we remark hernia amongst the most frequent complications. Mr. Esdaile (*Medical Gazette*) refers the disease, in a very large majority of cases, to hydrocele; we may, therefore, include this amongst its complications. Clot-Bey, after having observed a great number of complicated cases, says he never say a patient die of elephantiasis. Larrey, however, suspects "that by the incessant growth of the morbid mass, it may absorb, as it were, into itself, the vitality of the subject, causing death by consumption; or, perhaps, gangrene may invade the tumor, and the partial mortification of the tissues will destroy the patient." A fact of this kind seems to have come under the observation of Hendy. This kind of tumor seems not disposed to degenerate into cancer. Larrey, at least, never saw a case thus terminating. He, however, gives the case of Delacroix, originally related by Imbert de Lonnes, and commented on by Boyer (*Traite des Maladies Chirurgicales*, tom. 10), to express his doubts of its being a case in point, to prove the disease terminating in cancer.

He questions whether the unaided powers of nature be equal to the removal of the complaint in any instance; and thinks there is very little chance of a cure even from the ordinary resources of medicine, except, perhaps, at the very commencement. Nevertheless, he quotes Hendy's celebrated case (vide 22nd, case "Glandular Disease of Barbadoes"), where a man, affected with elephantiasis, awoke one morning having an abundant moisture about his thighs, which proved to be water effused through a fissure or crack in the diseased skin. A few months after this there was a recurrence of the evacuation, and the part was reduced almost to its normal volume.

As the writer details the structure in this disease, he is particularly clear and satisfactory. "The skin at first is not altered; it may even get thin from distention but it soon hardens; becomes thick and rugous; assumes sometimes the consistence of half-tanned leather, even to the thickness of three inches. It then acquires extraordinary extensibility. The epidermis grows thick and scaly. In most cases, the rete mucosum appears distinct. Chevalier saw the papilla exceedingly elongated, enlarged and prominent on the cutis. The normal texture disappears, and becoming hypertrophied, the tissue grows homogeneous, of a white color, of a brawny consistence, very hard, like scirrhus, without appearance or trace of fibres." "Cysts, as it were, are met with in the texture of the altered cutis, and within them there is a variable quantity of serous fluid." "The subcutaneous cellular tissue essentially participates in this hypertrophy by the thickening of its layers, as much more dense as they approach the cutis, and terminating by forming cellulofibrous cavities filled with albuminous or gelatinous fluid. This tissue, when indurated, frequently presents, like the cutis, a cartilaginous texture, and the matter secreted or accumulated within its cavities becomes sometimes so condensed as to form hard cretaceous and schirrhus-like nuclei. These nuclei of altered cellular tissue in some cases are isolated, constituting distinct tumors in the midst of the morbid mass, varying in their nature and volume."

The endemic nature of the disease, its peculiar mode of growth, progressing for years, and the very decided change of structure, would lead us to suppose it was not very amenable to medical treatment, and in this opinion most observers coincide; yet we cannot resist the evidence supplied by men worthy of credence, that not only has it been removed by suitable means, but that it has even disappeared completely from mere change of residence. Gibson, in the Institutes and Practice of Surgery, gives a case of this kind, and a somewhat similar one has been detailed by Larrey, yet he remarks that on the other hand, patients, even young, have come from the colonies to Europe, and, though aided by treatment, have experienced no amelioration.

"Antiphlogistic remedies, local bleeding, emollient poultices and fomentations, baths used in the beginning, frequently renewed and seconded by suitable diet and hygienic precautions, have procured happy results in the practice of Clot-Bey." Larrey doubts if they were lasting cures. He condemns general bleeding, unless employed with great reserve. This is in opposition to Rayer's practice, with which general bleeding entered as an important curative means, and who thought this opposition unfounded.

The writer has but little faith in the efficacy of sudorifics, and shares the incredulity of Delpech in this respect. "Avec quelle defiance," observes the accomplished surgeon of Montpellier, "ne doit-on pas accueillir des observations de guerison operee par des sueurs on telle autre evacuation augmentee, quand il s'agit d'une malade qui ne se complete qu' a la faveur d' un grand nombre d' annees!" Vomits, antimonials combined with mercury, mineral acids, the external application of dilute sulphuric acid, solutions of bi-chloride of mercury, of muriate of ammonia, and gradual and uniform compression and shampooing, are some of his many means used. In India, English surgeons have been using preparations of iodine; and a case is given in the *Lancet*, July, 1846, by Dr. Brett, attributing a cure to iodine.

The intermittent character of the fever so frequently accompanying the invasion of the disease, suggests the use of quinia, but we have no facts in reference to its efficacy, and in want of such, it is simply proposed for future trial. Some temporary advantage has been gained from arsenic, but it has never accomplished a cure. Setons, caustics, the actual cautery, punctures, scarifications, incisions, blisters, have all been proposed, used and vaunted, but on a close analysis of the supposed successful cases, the eye of science can often detect some fallacy, some error of diagnosis. In many instances, hæmatocele has been mistaken for elephantiasis scroti. "When the tumor is recent and as yet little developed, we may try with some hope of success local bleedings, emollient applications, sulphate of quinia, hydriodate of potash, mercurial frictions, methodical compression and shampooing." Should these fail, we have no resource except in extirpation. Even to this day, says Rayer, the question of the propriety or impropriety of extirpation is not settled. Indeed, we have a host of witnesses to the danger and frequent inefficacy of the operation. Alard assures us that those operated on have either become affected with elephantiasis elsewhere, or have sunk under some inflammatory affection of the viscera. Chopart opposed it from the danger of hemorrhage, yet surgeons seem now beginning to entertain less apprehensions of the operation itself and of its consequences.

Since Baron Larrey and Roux led the way in successful operation, others have performed it, and in the majority of instances with success. But above all, Esdaile of Calcutta has signalized himself. He has operated more than 160 times, and lays down the following rules for guidance: "When the constitution is good, and the patient not more than 40 years, and the tumor does not exceed fifty pounds in weight, it may be possible to save the testicles; but if the age and weight exceed these proportions, an effort should not be made to spare them, because the patient may die of hemorrhage during the attempt made to accomplish it, and even if he should not, the testicles may be found useless and require removal afterwards." As to the penis, it has always been spared, with one exception, by Mr. Esdaile, whatever might be the morbid mass removed. The mortality, he assures us, has been but five per cent., and in no instance has death been directly occasioned by the operation; and when it did occur, it was at a distance of many days, weeks or months afterwards, and took place from various morbid causes, not immediately connected with the operation. Mr. Esdaile is the writer who has so reprobated the employment of chloroform, for which he substitutes mesmerism, on the grounds that as the latter exalts, whilst the former depresses the nervous system, the peculiar applicability of this agent is sufficiently obvious.

In the operation, certain objects must be kept in view, although particular conditions of the tumor may occasionally require us to vary our proceedings. Thus, sufficient covering must be retained for the penis, testicles and their cords, the remainder of the tumor removed, and the several steps of the operation gone through with as much rapidity as is consistent with the preservation of the genital organs. Baron Larrey's method consisted, 1st, In two incisions, commencing at the orifice of the prepuce, obliquely carried under the testicles of each side; 2nd, Dissecting the tissues in the interval of the incisions; 3d, Removal of the diseased mass. In this manner also, Titley and Græfe operated. Clot-Bey substitutes for the oblique two semilunar incisions, extending from the pubis to the perineum. This mode of operation is termed "*L' extirpation sans anaplastie.*" "*Extirpation avec anaplastie*" consists in raising three flaps of healthy integument,—two lateral, of semilunar shape, and an intermediate one. These are to cover the penis, testicles and their respective cords. A third method, ascribed to Redlich, is, after removing of the tumor, to restore to the penis its original covering. Then there is Lenoir's method, characterized by an incision through the whole thickness of the scrotum, passing anteriorly and in the middle line; the penis is now exposed, and the testicles discovered, dissected and raised with their cords; then two lateral flaps are cut, and lastly the tumor removed by bold and rapid incision. The patient should be secured as in operation for stone; two assistants ought to have charge of his legs, and one or two of the tumor. Should this be very large, it is proposed to use mechanical means for raising it. "Before beginning operative proceedings, the tumor should be so raised as to allow the blood to return into the general circulation. Search should also be made for a hernia, and if such exist, previously reduce it; and in the operation, the sac should be opened, and tied afterwards at the external abdominal ring. Should the complication be hydrocele, instead of puncturing or incising it, excision should at once be made."

We now take leave of M. Larrey and his able report, with the conviction of having, from the short space allowed us, failed to present it in a light best suited to reflect facts and observations that must be read to be duly appreciated. We trust, however, our readers will see, even through the imperfect medium of an editorial notice, as much as may induce a further acquaintance with the report itself, the perusal of which will amply compensate for the required time and labor.

[In order to give a more adequate idea than can be conveyed by words alone of the magnitude of the growth which sometimes reaches the enormous size of 125 pounds and over, we have taken pains to have prepared the annexed figure, which accurately represents the original, somewhat reduced in size.]

ELEMENTS OF MEDICINE. By SAMUEL H. DICKSON, M.D. Philadelphia: Lea & Blanchard. 1859.

In the preface to this work, the author states that "it is intended as an aid to young men who have engaged in the study of medicine, and to physicians who have recently assumed the responsibilities of practice."

In this age of vain display and empty pretension, this unostentatious statement may give some a very inadequate notion of the merit of a work, which we think not only eminently calculated to answer the end proposed, but also well adapted to be profitable even to those who may deem themselves far in advance of their juniors in a knowledge of their profession. There pervades the volume an easy, unrestrained mode of expression, that cannot fail to carry the reader, almost imperceptibly to himself, over some of those stumbling blocks of science that so frequently in limine discourage the student. Nothing having a practical bearing is omitted. The various theories, under the head of etiology, that have influenced practice are brought under review, and with as much brevity as is consistent with the subject and the design of the work, are subjected to a searching analysis.

We cannot avoid expressing our commendation of the strictly logical accuracy with which the author reasons. This is almost a new feature in medical works; to the absence of which may be attributed the slow progress medicine has made in comparison with other departments of science.

Though we thus favorably notice this performance, yet we must pronounce it an unequal one. It is divided into two parts,—"General Pathology, and Special Pathology and Therapeutics." The former has our unqualified recommendation; the latter, with perhaps the section on fever excepted, does not realize the expectations the excellences of the former had led us to entertain. It would appear as if the author, with a view to render the work compendious, and thus place it within the reach of students, has done himself an injustice, by occasionally sacrificing to conciseness what is necessary to clear conception—graphic and full description; in short, where we want accurate delineation, we have but a hasty and imperfect sketch.

A man so capable of dealing with the abstruse things of the profession, as the perusal of the "General Pathology" will at once show, could with much more ease acquit himself equally well in "Special Pathology and Therapeutics."

On the whole, it will prove a most useful manual, which should not only be in the schools, but deserves a place in the library of every physician.

## Figures and Tables

**Figure f1:**